# Efficacy of ImageJ in the assessment of apoptosis

**DOI:** 10.1186/1746-1596-7-15

**Published:** 2012-02-06

**Authors:** Iman M Helmy, Adel M Abdel Azim

**Affiliations:** 1 Oral Pathology Department, Ain Shams University, Cairo, Egypt

**Keywords:** Apoptosis, Hep2 cell, Image analysis, NAF (nuclear area factor)

## Abstract

**Objective:**

To verify the efficacy of ImageJ 1.43 n in determining the extent of apoptosis which is a complex and multistep process.

**Study Design:**

Cisplatin in different concentrations was used to induce apoptosis in cultured Hep2 cells. Cell viability assay and nuclear image analysis of stained Hep2 cells were used to discriminate apoptotic cells and cells suspected to be undergoing apoptosis from control cells based on parameters such as nuclear area, circularity, perimeter and nuclear area factor (NAF), in association with visual morphology.

**Results:**

Image analysis revealed a progressive and highly significant decrease in nuclear area factor detected in apoptotic cells and in cells suspected of undergoing apoptosis compared to the control cells (P-values < 0.01). Some of the other studied parameters showed also the same trend. This decrease was assumed to indicate DNA loss. Image analysis results correlated positively and significantly with the results obtained by cell viability assay (R = 0.958, P-value = 0.042). NAF was the most reliable parameter in assessment of apoptosis.

**Conclusion:**

Nuclear area factor can be calculated using powerful free and open-source software. Consequently, a quantitative measure of apoptosis can be obtained that is linked to morphological changes. ImageJ 1.43 n may therefore provide a useful tool for the assessment and discrimination of apoptotic cells.

**Virtual slides:**

The virtual slide(s) for this article can be found here:

http://www.diagnosticpathology.diagnomx.eu/vs/5929043086367338

## Introduction

Apoptosis is an active process of self-destruction, whereby cells undergo physiological cell death. It occurs during development and regulation of tissue homeostasis or as a result of changes in environmental stimuli [1].

Induction of apoptosis is due to the absence of specific interactions between transmembrane receptors of the integrin family and extracellular matrix proteins [2]. The triggering of apoptosis requires the activation of specific enzymes called caspases [3], differently associated as inactive form with signal transduction complexes or with cellular organelles [1].

The rate of cell apoptosis is a significant parameter in many experiments involving cell cultures. Cell death kinetics can be measured by counting the number of cells and/ or area occupied on each culture dish by analyzing images taken at different moments of their evolution, apoptosis leads to condensation and fragmentation of cell bodies, producing regions populated with unstructured smaller objects [4].

Examining cells by microscopy has long been a primary method for studying cellular function. When cells are stained appropriately, visual analysis can reveal biological mechanisms [5].

Cells in advanced stages of apoptosis exhibit morphological changes characterized by nuclear and cytoplasmic condensation and cell fragmentation into membrane bound apoptotic bodies. These morphological changes can be observed by light microscopy and are correlated initially with large and subsequently, very small chromosomal DNA fragments [6]. Indeed, other morphological features of apoptosis occur in the absence of detectable DNA fragmentation or a decrease in DNA content [7].

Still, for most applications, image cytometry (automated cell image analysis) is strongly preferable to analysis by eye. In fact, in some cases image cytometry is absolutely required to extract the full spectrum of information present in biological images for many reasons such as: it yields many informative measures of cells, consistent quantitative measures for every image, measures each rather than producing a score for the entire image and finally is much less labor-intensive and higher-throughput [5].

Currently there are many systems available to an anatomic pathologist for the purpose of image analysis in nuclear morphometry. Many of these systems, however, require expensive software and hardware attachments for image acquisition, analysis and storage. Consequently, a cost-effective alternative for image analysis would be a welcome tool for pathologists and researchers alike. In this connection, "ImageJ" is a freely available java-based public-domain image processing and analysis program developed at the National Institutes of Health (NIH). At present, ImageJ's Macintosh platform counterpart, "NIH Image" is widely used in biologic research. A number of nuclear morphometric descriptions can be evaluated on stained sections after downloading specific plug-ins from the ImageJ website. The macros and plug-ins are available as source files and download to the ImageJ folder [8].

Image analysis of stained nuclei should be capable of revealing changes in chromatin organization and in DNA amounts during apoptosis. Such analysis should allow the detection of the early stages of apoptosis by associating slight changes in nuclear morphology with a decrease in DNA content [9].

There is a number of biochemical and image based essays for apoptosis that vary widely in complexity, specificity and cost [10]. It has previously shown that the nuclear area factor (NAF) can be an early indicator of cell morphological changes occurring during apoptosis [11]. Furthermore, calculation of NAF is relatively straightforward, and may be accomplished with a number of image analysis programs.

As long as the cell nucleus remains somewhat intact and the DNA does not become completely fragmented, calculation of NAF should remain a valid marker of apoptotic changes, even though the greatest extent of these changes may occur early in the apoptosis process [10]

A systematic undercount of nuclei by image analysis method may produce variation, due in part to the inability to accurately determine a nuclear count when nuclei touch one another. Hence, the image analysis method required thin sections to minimize this problem [12].

In a previous study, there was a disparagement for the public image analysis software program ImageJ 1.37 v, the author proclaimed that while ImageJ was free available, it did not include all of the preanalysis filters and features which are often essential for separating cells before calculating NAF or other measurements especially in cases where cells may aggregate or clump in vitro either in normal conditions or after apoptotic stimuli, and tools available in Image Proplus for separating and splitting objects, are useful for calculating NAF [10].

In concept, calculation of NAF could as easily be used with nuclear dye such as hematoxylin or Giemsa, which we used here. A benefit of these blue dyes is that they allow for simultaneous staining of the same cells with independent markers for apoptosis such as FITC-based TUNEL [10].

NAF was calculated as the product of area (in pixel^2^) roundness. For calculating NAF from ImageJ data, since roundness was not an available function, as an alternative circularity was used.

The existence of a variety of apoptosis inducers and of variable expression according to the cell type considered has led to a reconsideration of the morphological criteria for the detection of apoptosis. Even in studies using in situ labeling of DNA strand breaks as a marker, there is a heavy reliance on morphological features for the identification of apoptotic nuclei. This study was conducted to proof the efficacy of ImageJ 1.43 n as open source software in assessment of apoptosis which is a complex and multistep process.

## Material and methods

### A. Cell line

**- **Squamous cell carcinoma cell line Hep2 used in this research work was kindly supplied from Cell Culture Department-VACSERA, Egypt. Hep2 cells were grown in Minimum essential medium modified with Hank's salts (MEM-H) supplemented with 10% fetal bovine serum (FBS), 2 mM glutamine and sodium bicarbonate. The Hep2 cells were treated with Cisplatin (10 μgm, 5 μgm and 2.5 μgm) for 24 hours. Some Hep2 cells remain untreated and used as control.

### B. Determination of Cytotoxic Effect of Cisplatin on HEp2 Cell Line

#### 1. Cellular viability assay

**- **The treated dead cells detected were discarded by plate washing using phosphate buffer saline PH 7.2 ± 0.2, secondly the cells were fixed with 10% formalin for 1 hour then fixative was discarded. Plates were washed as previous, and then stained with 0.05% crystal violet solution in 20% ethanol for 10 minute. The extracellular dye was removed by thoroughly rinsing of the cell monolayer using tap water. The remaining cell-attached dye was dissolved in 0.1% acetic acid solution in 50% ethanol. The optical density was read at 540 nm using ELISA reader. Cell viability percentage was evaluated as the percentage of the mean value of optical density of treated wells divided by the mean value of optical density of negative cell control.

#### 2. Morphometric analysis

**- **Treated Hep-2 cells were separately stained with heamatoxylin and eosin, and Giemsa stains to detect the histomorphological changes in the Hep-2 cells caused by the cytotoxic drug. For Giemsa staining, the slides were fixed with methyl alcohol for 3-5 minutes, immersed in diluted Giemsa stain for 10-20 minutes and then washed with buffered water (PH 6.8). After that, slides were dried, mounted with Canada balsam and cover slips were placed.

**Figure 1:**
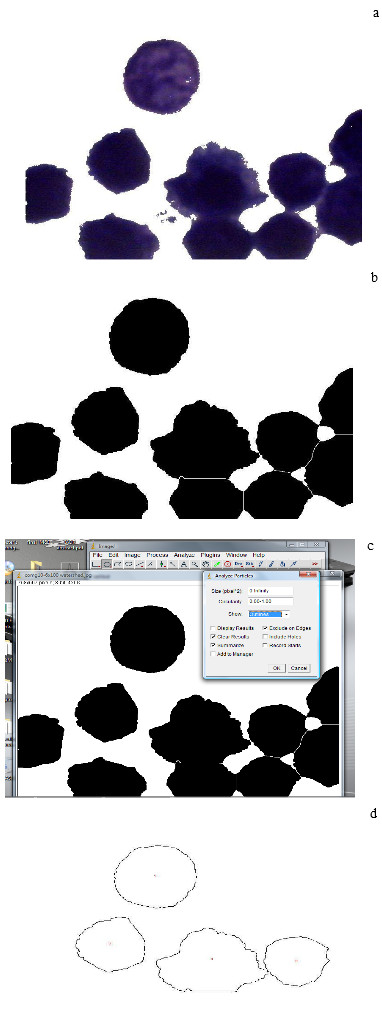
**(a) Giemsa stained section of Hep2-treated cells showing sampling of the color to be thresholded**. **(b) **Thresholding of the color and watershed of 8-bit image. **(c) **Analyzing particles after excluding edges. **(d) **Drawing outlines and automatic numbering of the desired objects.

The surface area, perimeter (the length of the outside boundary of the selection) and circularity of the nuclei were automatically measured and then NAF was calculated by the product of nuclear area and circularity (steps of measurements are shown in Figures 1a, b, c and 1d).

In ImageJ 1.43 n, the formula for circularity is 4π (area/perimeter^2^). A value of 1.0 indicates a perfect circle, which can be associated with apoptosis. Note that the circularity field was first added to ImageJ ver1.35e. As the circularity value approaches 0.0, it indicates an increasingly elongated polygon. The user notes for ImageJ software states that measuring circularity may lead to invalid values for very small particles. These aberrant readings can be eliminated by excluding particles that are below a specific minimum area [10].

### Statistical analysis

Statistical analysis was performed using (SPSS 17). ANOVA test was achieved to test the differences in the morphometric measurements in the control cells and cells undergoing apoptosis induced by the effect of Cisplatin. Then, it was followed by Tukey post-hoc test. Correlation between cell viability test and NAF was achieved. Statistica7 software was used to generate the graphs of statistical data.

## Results

**Figure 2:**
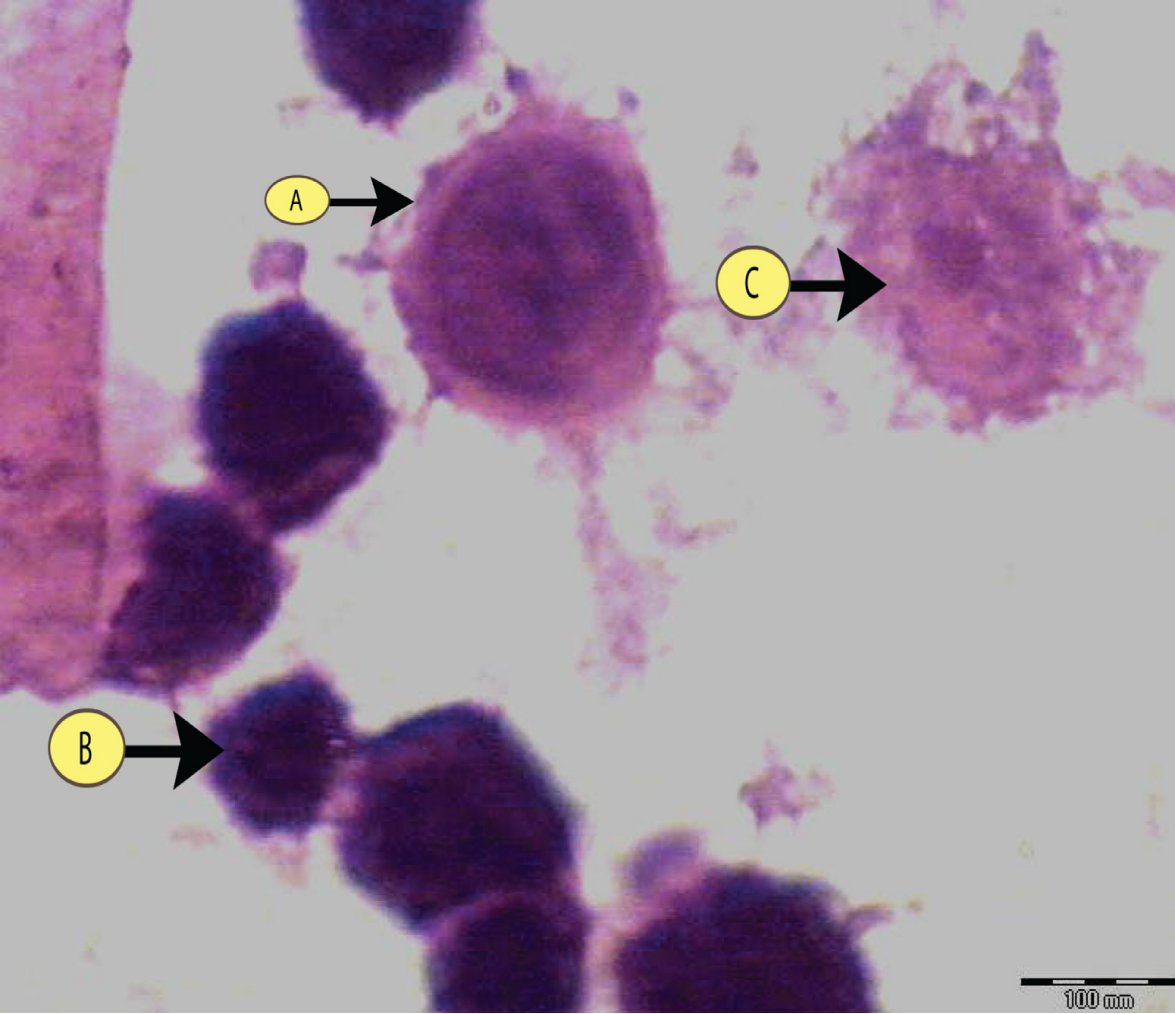
**H&E stained section of Hep2 cells showing the 3 phenotypes detected in culture with different chromatin packing**.

**Figure 3:**
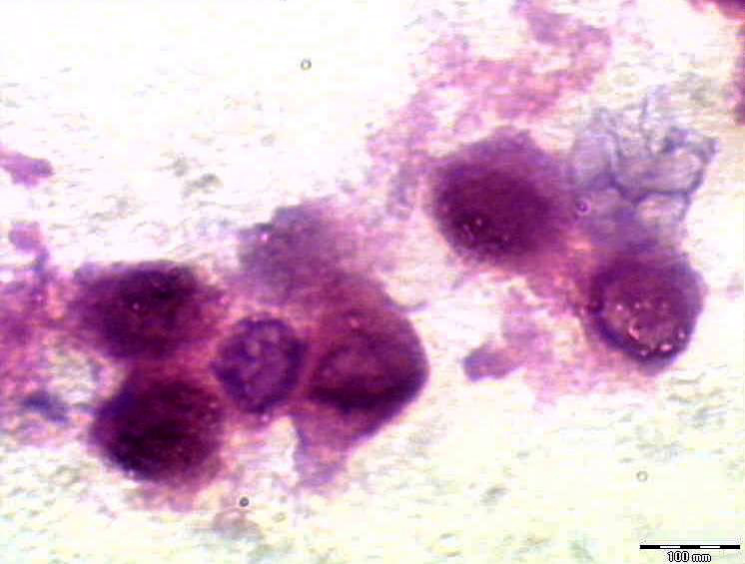
**H&E stained section showing control cells in culture**.

Three phenotypes were detected in the culture differing in their chromatin packing states and intranuclear distribution, the phenotype (I) was characterized by a few small chromatin granules in a homogeneously distributed, delicate chromatin background (Figure 2a). Another phenotype (II), designated as suspected apoptosis, exhibited larger areas of condensed chromatin and a reduced nuclear area compare to type I nuclei (Figure 2b). In the third phenotype (III) the nuclei clumps of condensed chromatin distributed at the nuclear periphery. Entire III nuclei frequently appeared condensed into clusters of round corpuscles (Figure 2c). The apoptotic nuclei chosen for measurements showed no or minimum signs of nuclear envelope disruption. Figure 3 shows control cells for comparison.

A negative correlation between the concentration of Cisplatin and cell viability percentage (as drug concentration decrease the viability percentage increase) from concentration 10 μgm to 2.5 μgm, then with any increase in concentration there was no increase in the number of apoptotic cells.

**Figure 4:**
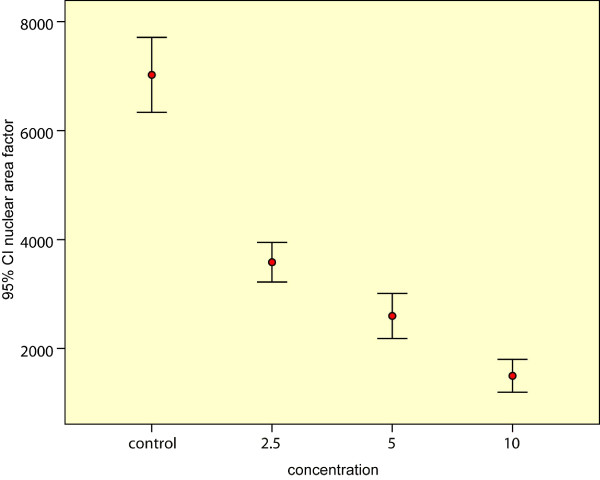
**Error bars showing the effect of different concentrations on the nuclear area factor**.

For calculations using ImageJ, both the average area and circularity of Hep2 treated cells decreased compared to control cells, resulting in dramatic reduction of NAF from 7,023 in control cells to 1,497 in Hep2 cells treated with Cisplatin (table 1). Since, the NAF is the product of area and circularity, a decrease in NAF was observed using ImageJ analysis (Figure 4).

**Table 1:** Descriptive Statistics for Nuclear Area Factor at Different Concentrations*

	N	Mean	Std. Deviation	Std. Error
control	27	7,023^a^	1,740	335
2.5	400	3,583^b^	3,696	185
5	345	2,598^c^	3,912	211
10	292	1,497^d^	2,622	153

* Means with different superscripts are statistically different at the level of a 0.05

**Figure 5:**
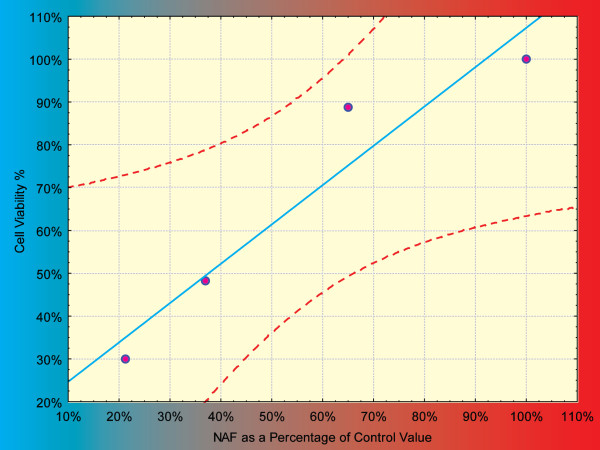
**Plotting the NAF with cell viability with the trend line and confidence interval lines**.

There was a significant decrease in the nuclear area factor detected in the apoptotic cells and cells undergoing apoptosis, induced by different concentrations of Cisplatin, compared to the control cells (P value < 0.01), this decrease denoting DNA loss. There was also a significant positive correlation between the results of cell viability assay and morphometric assessment of apoptosis by ImageJ (Figure 5) (R = 0.958, P-value = 0.042).

## Discussion

Apoptosis proceeds through typical alterations of nuclear morphology [13] characterized by a progressive condensation of chromatin that induces nuclei to shrink into little single balls or cluster of grapes [14]. Thus a nuclear stain such as heamatoxylin can well be chosen to evidence alterations in nuclear morphology [1].

The first apoptotic stage is compatible with an initial step of caspase-3 activation in the cytoplasm, characterized by normal nuclear morphology faintly stained by heamatoxylin. The second stage, characterized by cytoplasm and nuclear over-staining, may represent the step of increasing caspase-3 activation in the cytoplasm and its presence into the nucleus. The third stage identified fits in well with the typical picture of the late apoptotic phase, characterized by nuclear envelope fragmentation [15].

ImageJ software is freely available on the web, and does not allow particle analysis of color images. Therefore, images were converted to 8-bit grayscale, and then thresholded. From these thresholded images, ImageJ then outlines and numbers each object. ImageJ also has the function of "cleaning edges", thus cells on the edge of the image and cut by the image border can be excluded from the final analysis. The old version of ImageJ did not have a "split" function used to separate closely touching cells. Thus cells that cannot be separated by thresholding remain as larger groups, and also need to be excluded from the final analysis, but this drawback was enhanced in the recent version. The new version of ImageJ has the watershed function that can split the closely touching cells. Thus, cells that cannot be separated by thresholding can be split by the watershed function. The larger number of available functions for cell counting added to the new version of ImageJ results in more objects that can be included in the count.

In the old version of ImageJ, while objects can be theoretically separated using thresholding, it was a destructive process, which makes all objects smaller when thresholding is increased, in the new version there is the option of automatic thresholding using sample of the desired color, and consequently thresholding is more accurate.

For calculations using ImageJ, both the average area and circularity of Hep2 cells decreased compared to control cells, resulting in dramatic reduction of NAF from 7,023 in control cells to 1,497 in Hep2 cells treated with Cisplatin. Since, the NAF is the product of area and circularity, a decrease in NAF was observed using ImageJ analysis.

The technique reported here may have utility for measuring cell morphological changes such as those occurring in cancer. For example, the nuclear morphometry measures of mean nuclear area, mean nuclear elongation factor, and mean nuclear regularity factor were used as potential predictors for clinical outcomes in localized renal carcinoma [16]. However, in a consensus report of prognostic factors in prostate cancer, it was deemed that factors such as nuclear circularity and nuclear area required further studies before routine use could be recommended [17].

In short, NIH Image/ImageJ delivers a lot despite being a freeware. Knowledge of its potential uses will go a long way in establishing objective, reproducible, cost-effective and timesaving methods of automated image-based IHC evaluation and morphometry for histopathologists.

## Competing interests

The authors declare that they have no competing interests.

## Authors' contributions

I H participated in the design of the study, coordination, sequence alignment and drafted the manuscript. A A performed the statistical analysis and reviewing the manuscript. All authors read and approved the final manuscript.
